# An Extended Multilocus Sequence Typing (MLST) Scheme for Rapid Direct Typing of *Leptospira* from Clinical Samples

**DOI:** 10.1371/journal.pntd.0004996

**Published:** 2016-09-21

**Authors:** Sabrina Weiss, Angela Menezes, Kate Woods, Anisone Chanthongthip, Sabine Dittrich, Agatha Opoku-Boateng, Maimuna Kimuli, Victoria Chalker

**Affiliations:** 1 Public Health England (PHE), National Infection Service (NIS), London, United Kingdom; 2 European Programme for Public Health Microbiology (EUPHEM), European Centre for Disease Prevention and Control (ECDC), Stockholm, Sweden; 3 Lao-Oxford-Mahosot Hospital-Wellcome Trust Research Unit (LOMWRU), Vientiane, Lao PDR; 4 Centre for Tropical Medicine and Global Health, Nuffield Department of Medicine, University of Oxford, Oxford, United Kingdom; 5 Foundation for Innovative New Diagnostics (FIND), Geneva, Switzerland; Institut Pasteur, FRANCE

## Abstract

**Background:**

Rapid typing of *Leptospira* is currently impaired by requiring time consuming culture of leptospires. The objective of this study was to develop an assay that provides multilocus sequence typing (MLST) data direct from patient specimens while minimising costs for subsequent sequencing.

**Methodology and Findings:**

An existing PCR based MLST scheme was modified by designing nested primers including anchors for facilitated subsequent sequencing. The assay was applied to various specimen types from patients diagnosed with leptospirosis between 2014 and 2015 in the United Kingdom (UK) and the Lao Peoples Democratic Republic (Lao PDR). Of 44 clinical samples (23 serum, 6 whole blood, 3 buffy coat, 12 urine) PCR positive for pathogenic *Leptospira* spp. at least one allele was amplified in 22 samples (50%) and used for phylogenetic inference. Full allelic profiles were obtained from ten specimens, representing all sample types (23%). No nonspecific amplicons were observed in any of the samples. Of twelve PCR positive urine specimens three gave full allelic profiles (25%) and two a partial profile. Phylogenetic analysis allowed for species assignment. The predominant species detected was *L*. *interrogans* (10/14 and 7/8 from UK and Lao PDR, respectively). All other species were detected in samples from only one country (Lao PDR: *L*. *borgpetersenii* [1/8]; UK: *L*. *kirschneri* [1/14], *L*. *santarosai* [1/14], *L*. *weilii* [2/14]).

**Conclusion:**

Typing information of pathogenic *Leptospira* spp. was obtained directly from a variety of clinical samples using a modified MLST assay. This assay negates the need for time-consuming culture of *Leptospira* prior to typing and will be of use both in surveillance, as single alleles enable species determination, and outbreaks for the rapid identification of clusters.

## Author Summary

Leptospirosis is a zoonotic disease with more than 1 million cases per year globally and epidemics are increasingly reported. In this setting rapid typing is essential to identify potential clusters and transmission pathways. Typing of bacteria commonly requires bacterial isolates but culturing *Leptospira* is difficult and time consuming and requires invasive samples, such as blood or cerebrospinal fluid. We modified an existing typing scheme to lower the limit of detection and were able to amplify and sequence alleles directly from clinical specimens. Samples included blood (whole blood, serum, or buffy coat) and urine from patients diagnosed by PCR with leptospirosis between 2014 and 2015 in the United Kingdom and the Lao Peoples Democratic Republic. Using the sequences in phylogenetic analysis we identified the predominant *Leptospira* species in both countries as *L*. *interrogans*. With its increased sensitivity the modified assay allows for typing and species determination of *Leptospira* directly from blood or urine. It will be of use during epidemics and outbreaks for rapid identification of clusters and can support surveillance without the need to culture fastidious isolates.

## Introduction

Leptospirosis is a zoonotic disease caused by pathogenic species of *Leptospira* that can be carried naturally by most mammalian species [1–3]. Transmission to humans most commonly occurs via direct animal contact or via water contaminated with animal urine [2, 4]. Symptoms range from a mild febrile illness to severe disease with pulmonary haemorrhage or central nervous system involvement [3, 5]. In its early stages leptospirosis resembles many other febrile illnesses, hampering clinical diagnosis. The highest disease burden is in tropical low and middle income countries, driven by high humidity, close human-animal contact, and inadequate sewage disposal and water treatment [3]. Annual worldwide case number was estimated at around 1 million with the majority of cases and death occurring in tropical regions [6]. Despite these relatively high numbers the epidemiology of leptospirosis is not well understood. Epidemics in humans and animals are increasingly reported and are often related to natural events like floods [3, 7]. In these settings rapid typing is essential to identify potential clusters and transmission pathways.

The gold standards for laboratory diagnosis of leptospirosis are culture or a four-fold rise in antibody titre between admission and convalescent samples by the microscopic agglutination test (MAT). Culture of *Leptospira* spp. is time consuming and diagnosis by MAT is retrospective by nature, hence both methods have disadvantages as diagnostic tools. To enable early detection several quantitative real-time PCR assays have been developed, some of which allow for species distinction [8–20].

Three MLST schemes are currently hosted by the public MLST database [21–23], two of which have been tested directly on clinical samples from humans [24–26]. Only two studies tried to amplify all seven loci and showed that MLST is possible directly from serum and whole blood. However the bacterial load required was high (~5x10^4^ leptospira/mL) with only 21% and 5% or 10% success rates for partial and full profiles, respectively [24, 26]. The objective of this study was to develop an assay based on a published MLST scheme that lowers the limit of detection (LoD) to enable rapid provision of typing data directly from patient specimens whilst minimising costs for subsequent sequencing [22].

## Methods

### Ethics statement

Specimens included in the study were not collated specifically for this study. Specimens included those within a collection of specimens submitted to the Public Health England Leptospira Reference Laboratory received routinely for *Leptospira* testing, identification of infecting species, confirmation of infection and for epidemiological investigation. Specimens were anonymised prior to testing. IRB board approval was not required as this involved routine specimens submitted for *Leptospira* testing by MLST as a secondary test for confirmation of infection and species identification and for the provision of epidemiological information.

### Bacterial isolates, patient samples, and DNA extraction

The protocol was validated on 25 isolates from the WHO recommended Serovar panel (data in S1 Table) which is currently used for serological diagnostic and serovar identification. The assay was tested using 104 clinical specimens (45 serum, 6 whole blood, 13 buffy coat, 40 urine) from the UK (n = 35) and the Lao PDR (n = 69), (Mahosot Hospital Microbiology Laboratory, Vientiane). For initial laboratory diagnosis samples were tested with a triplex qPCR assay targeting the 16S rRNA gene (*rrs*) containing three different probes to distinguish between pathogenic, intermediate and environmental strains [27]. Using this assay, 44 samples (23 serum, 6 whole blood, 3 buffy coat, 12 urine) tested positive for pathogenic *Leptospira* spp. and 15 were negative. In addition, 16 environmental and 29 intermediate *Leptospira* spp. positive samples were included in the panel as negative controls as they should not be detected by the MLST scheme. Testing was performed blinded. A detailed list of pathogenic *Leptospira* spp. positive samples and origin can be found in the table in S2 Table.

For each sample, 200 μl sample material was used for extraction. For urine samples from Lao PDR 1.5 mL was spun down at 14000 rpm for 15 minutes before it was used for extraction. DNA from bacterial isolates and Lao PDR samples was extracted using the QIAmp DNA Mini Kit (Qiagen, Germany) according to manufacturer‘s instructions. DNA from UK samples (C1-C10) was extracted on the MagNA Pure Compact (Roche, Germany) using the DNA_Bacteria Protocol. These samples and bacterial isolates were eluted once in 50 μL nuclease-free water. Samples from Lao PDR were eluted twice in 50 μl nuclease-free water to reach a final volume of 100 μL. UK samples P1-P25 were extracted on the EZ1 investigator platform (Qiagen, Germany) and eluted in 120 μL.

### MLST scheme and sensitivity analysis

MLST was performed based on a published scheme targeting seven loci (*glmU*, *pntA*, *sucA*, *tpiA*, *pfkB*, *mreA*, *caiB*) of seven pathogenic *Leptospira* species (*L*. *alexanderi*, *L*. *borgpetersenii*, *L*. *interrogans*, *L*. *kirschneri*, *L*. *noguchii*, *L*. *santarosai*, *L*. *weilii*) [22]. The protocol was adapted by using the HotStar Taq Master Mix (Qiagen, Germany) in a 20 μl reaction including additional 100 nmol MgCl_2_ for locus 4 (*tpiA*) only, 5 pmol of each primer, and 40–60 ng DNA. For clinical samples, 5 μl DNA extract was used. Cycling conditions remained unchanged, except for additional initial 15 minutes incubation at 95°C to activate the enzyme. Further to the published protocol, nested primers were designed for all loci in the original MLST scheme (Table 1) to improve the LoD. Primer sequences were based on multi-sequence alignments of all serovars available in this study. To facilitate downstream sequencing primers were extended with M13 anchor primers.

**Table 1:** MLST nested primers for all seven loci designed to accompany the MLST scheme by Boonsilp *et al*., 2013 [22].

locus	primer name	sequence (5'–3')	PCR product (bp)
***glmU***	1-glmU-2F_M13	TGTAAAACGACGGCCAGT CGYATGAAAACGGATCAG	598
	1-glmU-2R_M13	CAGGAAACAGCTATGACC GGAAGRTARTATTCDCCCTG	
***pntA***	2-pntA-2F_M13	TGTAAAACGACGGCCAGT ATTTATYTVGGRATGTTYCA	607
	2-pntA-2R_M13	CAGGAAACAGCTATGACC GATTTCATRTTATCYACRAT	
***sucA***	3-sucA-2F_M13	TGTAAAACGACGGCCAGT GCSGGTRATCATCWBATGG	552
	3-sucA-2R_M13	CAGGAAACAGCTATGACC GRAAWCCYTTYGCAAGATC	
***tpiA***	4-tpiA-2F_M13	TGTAAAACGACGGCCAGT ATTTCYTTACGAATRAAAGARTG	555
	4-tpiA-2R_M13	CAGGAAACAGCTATGACC CMCATTCGATYMRAGAAAA	
***pfkB***	5-pfkB-2F_M13	TGTAAAACGACGGCCAGT GTYGTATCGATCGSYTTC	540
	5-pfkB-2R_M13	CAGGAAACAGCTATGACC YYCCSGAAGAYAASGGWCAT	
***mreA***	6-mreA-2F_M13	TGTAAAACGACGGCCAGT CRRGAAGYRGTGGATCAGG	568
	6-mreA-2R_M13	CAGGAAACAGCTATGACC CKATCCTTACTYTCRTARCT	
***caiB***	7-caiB-2F_M13	TGTAAAACGACGGCCAGT CTTKCTTCRATYTTGGCG	589
	7-caiB-2R_M13	CAGGAAACAGCTATGACC AMCGATATGTWAYMGGRGTT	

The nested PCR was performed in 20 μl reaction using 5 pmol of each primer and 2 μl of the first-round PCR product. Cycling conditions were as follows: 10 min at 95°C, 5 cycles of 30 sec at 95°C, 30 sec at 46°C, 30 sec at 72°C. This was followed by 10 cycles with the annealing temperature increasing by 1°C per cycle and 20 cycles with an annealing temperature of 56°C. The final extension period was 7 min at 72°C. To avoid contamination different processes were performed in physically separated rooms. For detection of possible cross-contamination between samples that could occur during transfer of the amplicon from first to second round PCR non-template controls were included in all experiments and handled last. Further, only one sample was opened at a time and stringent cleaning measures were applied after each experiment.

To compare the detection limits serial dilutions of six DNA extracts from *Leptospira* isolates (Serovars Canicola, Grippotyphosa, Copenhageni, Hardjo, Mini, Pyrogenes) were tested using the original typing scheme and the second round PCR of the modified assay. Initial DNA concentration was 4 ng/μl, corresponding to 800,000 copies of genomic DNA (gDNA) or 8 x 10^5^ organisms (calculations based on the size of the genome of *L*. *interrogans* strain Fiocruz L1130 (4.6 Mb); 1 genome is ~5 fg). Serial dilutions were tested from 10^−2^ to 10^−5^ and PCR products were visualised on 2% agarose E-gels (Thermo Fisher Scientific, USA). In addition, 15 patient specimens (P1-P15) were tested with the modified assay first and second round PCRs.

### Sequence and phylogenetic analysis

PCR products were purified on an automated liquid handling robot (Biomek NXP) using Ampure XP paramagnetic beads (Beckman Coulter, USA). Sanger sequencing was carried out on the Applied Biosystems 3730XL Genetic Analyser (Thermo Fisher Scientific, USA). Sequences were assembled, edited, and trimmed using BioNumerics version 6.1 (Applied Maths NV). Sequence types (ST) were assigned by BioNumerics using allelic profiles in the order *glmU-pntA-sucA-tpiA-pfkB-mreA-caiB*. The same order was used to concatenate sequences for phylogenetic analysis. All new sequences have been submitted to the leptospira MLST database (http://pubmlst.org/leptospira/).

For species assignment sequences from all patient samples were included in phylogenetic analyses along with isolates from the WHO panel for which the species are known. Sequences were aligned in seaview4 [28] and used to construct maximum likelihood trees in *MEGA* version 6 [29] using the best suitable and available model for each alignment as determined by jModeltest [30].

## Results

The modified scheme allowed for amplification of all pathogenic *Leptospira* species covered by the scheme and represented in the WHO serovar panel (25/28). Two had new ST assigned (allelic profiles serovar Saxkoebing strain Mus 24: 24-69-30-35-37-26-51 [ST 219]; serovar Shermani strain 1342 K: 57-53-47-49-79-61-43 [ST 220]; data in S1 Table).

### Clinical samples

Fifteen clinical specimens (P1-P15) were tested using the first-round MLST assay and none gave a positive result. Applying the improved nested MLST assay five of these yielded at least one amplified locus; two samples gave full allelic profiles (P1 and P12).

In total, using the improved nested assay on 44 clinical samples PCR positive for pathogenic *Leptospira* species, 22 yielded a result in at least one allele detected that could be sequenced (50%). Full allelic profiles were obtained from 10 (23%) specimens, and partial allelic profiles from 12 specimens (27%, Table 2). No nonspecific amplicons were observed in any of the clinical samples. All negative control samples (including those positive for environmental and intermediate *Leptospira* species) were negative by MLST.

**Table 2:** Results of nested MLST PCR for clinical specimens by sample type and full/ partial allelic profiles.

MLST result	Bufffy coat	Serum	Urine	Whole blood	Total
**Full profile**	2	2	3	3	**10**
**Partial profile**	1	7	2	2	**12**
**No amplicon**	0	14	7	1	**22**
**Total**	**3**	**23**	**12**	**6**	**44**

Out of the twelve positive urine specimens, three gave full allelic profiles (25%), and two a partial profile (4 and 5 loci). In total, eleven new alleles were detected and five of the specimens revealed allelic profiles representing new ST. Despite several attempts three samples resulted in ambiguous nucleotides in sequences of two (L29, *sucA* and *caiB*) and one (C4 and P8, *pfkB*) loci. No numbers could be assigned to those alleles. The locus that was amplified most often from clinical samples was *caiB* (19/44, 43.2%), followed by *glmU* (18/44, 40.9%) (data in S3 Table).

### Comparing nested and original MLST scheme

Using the nested approach it was possible to lower the LoD of the assay. The minimum DNA concentration for simultaneous detection of all loci (42 PCRs) using the nested MLST scheme was 4x10^-4^ ng, corresponding to 80 copies of genomic DNA (gDNA; S1 Fig). In contrast, after the first round of amplification weak bands were visible for only eight loci (8/42, 19%). When using eight gDNA copies per reaction in the nested assay only two PCRs did not yield a detectable product (strain Hardjoprajitno /*pntA* and Salinem /*pfkB)* while no product was detectable using the first round PCR only.

### Phylogenetic analysis

**Fig 1:**
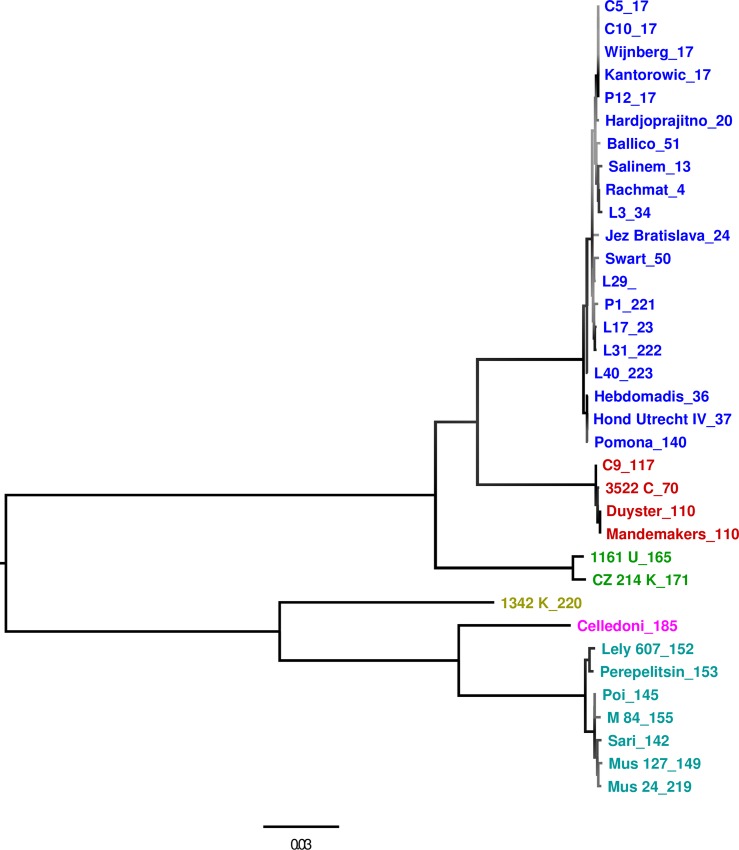
Maximum likelihood phylogenetic tree based on concatenated MLST sequences. All species defining branches are fully supported by 500 bootstraps. Bar represents substitutions per site. Tips are labelled with *strain_ST* (WHO panel) or *sample ID_ST* (clinical samples) and coloured by species: marine = *L*. *interrogans*, red = *L*. *kirschneri*, green: *L*. *noguchii*, yellow: *L*. *santarosai*, pink: *L*. *weilii*, light blue: *L*. *borgpetersenii*. P = 2015, C = 2014, both UK; L = Lao PDR, 2014.

For species assignment sequences from all patient samples were included in the phylogenetic tree along with isolates from the WHO panel for which species are known (S1 Table). A maximum likelihood tree showing all samples for which a full allelic profile could be obtained is shown in Fig 1. Trees based on separate alleles, are in concordance with the full-profile tree (S2 Fig). *L*. *interrogans* was the most frequently detected species in 17 samples (17/22, 77%). Table 3 shows the different species detected in each country.

**Table 3:** Species assigned to clinical specimens based on phylogenetic analysis.

Species	UK	Lao PDR	Total
***L*. *borgpetersenii***		1	**1**
***L*. *interrogans***	10	7	**17**
***L*. *kirschneri***	1		**1**
***L*. *santarosai***	1		**1**
***L*. *weilii***	2		**2**
**Total**	**14**	**8**	**22**

## Discussion

Using the developed nested amplification approach presented in this study it was possible to increase the MLST assay’s analytical sensitivity and obtain typing information of pathogenic *Leptospira* species directly from a variety of clinical samples. The developed assay is based on an established MLST scheme supported by a public website (http://leptospira.mlst.net/) and it will therefore not negatively impact comparability of already typed leptospires. The simplified PCR setup along with the anchor primers incorporated in the nested assay enables sequencing using two primers for all loci which will reduce costs. No nonspecific amplification was observed in any of the clinical samples. Consequently, in resource-limited settings where quantitative real-time PCR facilities are not available, the assay (or defined loci only) may be a useful diagnostic tool when applied with all necessary precautions to avoid cross-contamination between samples.

Sample numbers in the presented study are too low to make any inferences as to which specimen type is most promising for molecular typing. Success rates between different samples varied between 40–100%. The highest proportion of full allelic profiles was obtained from buffy coat (2/3) and whole blood (3/6), followed by urine (3/12). Due to the dynamics of the disease *Leptospira* may be found in blood or urine at different time points [8, 31]. Consequently, choice of specimen type and sampling time post symptom onset may prove critical for molecular MLST determination direct from specimens. In addition, as for any PCR based assay, detection is influenced by the genomic sequence of the strain present. Most primers used in the modified typing scheme were degenerated to account for sequence differences between the different strains, leading to variable specificity. Samples used for the present study were extracted using different platforms and elution volumes. However, all extracts were tested using the same diagnostic qPCR method and there does not appear to be a correlation between the original CT values and whether full or partial profiles were obtained (data in S3 Table). Similarly, there was no correlation between sample type or *Leptospira* species and successfully amplified locus. Interestingly, the locus that performed best in the nested assay (*caiB*) was the least reliable in a study from Argentina using the unmodified MLST scheme [26]. Overall, using the nested approach the success rate of detecting full or partial profiles could be improved by more than two fold when compared to previous studies applying the original MLST scheme directly on clinical specimens [24, 26].

Typing results of samples from the WHO serovar panel are 100% concordant with previously published results. Of note, the panel does not include an isolate of *L*. *alexanderi* and none of the clinical samples turned out as such. Boonsilp *et al*. (2013) characterized 325 isolates that resolved into 190 different ST and showed that *L*. *alexanderi* is detected by the original MLST scheme [22]. All loci represent conserved genes and the nested primers fit a representative sequence of *L*. *alexanderi*. It hence can be assumed that the nested assay would detect *L*. *alexanderi*, enabling it to detect all pathogenic *Leptospira* species, as well as ST that could not be tested for in the present study.

Single alleles amplified from clinical specimens allow for species determination when used in phylogeny, opening up the possibility for the assay to support surveillance. Currently, most human leptospirosis cases are not identified to species level, so it is difficult at this point to draw any further conclusions from the presented results. A recent survey conducted in Southeast Asia identified four pathogenic species in native rodents: *L*. *weilii*, *L*. *kirschneri*, *L*. *interrogans* and *L borgpetersenii*, the latter being the most prevalent [32]. This is consistent with the findings of our study. Similarly, in the UK and Europe, *L*. *interrogans* was identified in indigenous rodents [33, 34]. The variety of species found in the UK patients might be attributable to the fact that many cases in the UK are diagnosed in returning travellers. Of the 34 cases diagnosed in the UK, 15 reported a travel history (44%). Of these, 9 (26%) had travelled to South East Asia (Malaysia, Thailand and Indonesia). One case found to be infected with *L*. *weilii* had travelled to Thailand and one case infected with *L*. *santarosai* reported travel to Central America. The ability to obtain typing data directly from clinical specimens is ideal for pathogens that are difficult and slow to isolate in culture. The use of direct typing on urine specimens allows for non-invasive sampling and in some cases the provision of typing information in the absence of data from blood samples. One patient was positive for pathogenic *Leptospira* spp. in both serum and buffy coat by qPCR. MLST in this patient yielded a full profile from buffy coat, but only a partial profile (5 loci) from serum. While this is consistent with our finding that success rates for amplifying MLST loci were higher in buffy coat than in serum it has to be interpreted with caution due to low sample numbers.

Despite several attempts one sample resulted in ambiguous nucleotides in two loci (L29) and two samples in one locus (C4 and P8). This could indicate active infection with more than one strain. Another possibility is that more than one copy of the gene is present in the genome, as has been shown for the *mompS* gene of several *Legionella* strains [35].

In summary, the reported improved MLST assay represents a fast and specific tool for typing *of Leptospira* direct from clinical specimens, including non-invasive samples such as urine. It may be of use during epidemics and outbreaks by enabling rapid identification of *Leptospira* species and MLST types without the inherent delay involved in *Leptospira* culture.

## Supporting Information

S1 Table WHO recommended *Leptospira* serovar panel. Strain information was obtained from KIT Leptospirosis Reference Centre.
(XLSX)

S2 Table Specimens PCR positive for pathogenic *Leptospira* species. (XLSX)

S3 Table Allelic profile, species and CT value of diagnostic qPCR for MLST positive samples. (XLSX)

S1 Fig E-gel pictures of PCR products from first round and nested PCR of selected *Leptospira* strains in different dilutions. 1: Sari, 2: Wijnberg, 3: Hardjoprajitno, 4: Duyster, 5: Hond Utrecht IV, 6: Salinem. Dilutions (genomic copy numbers): A: 10E-2 (8000), B: 10E-3 (800), C: 10E-4 (80), D: 10E-5 (8). Dilutions are separated by one empty gel pocket. Each sample is applied in the following order: *glmU*-*pntA*-*sucA*-*tpia*-*pfkB*-*mreA*-*caiB*. DNA ladder size from top to bottom: 2000, 800, 400, 200,100 basepairs.
(PDF)

S2 Fig Phylogenetic trees including WHO recommended *Leptospira* serovar panel and clinical samples based on separate alleles. Bar represents substitutions per site. Tips are labelled with sample_ST and coloured according to species: marine = *L*. *interrogans*, red: *L*. *kirschneri*, green: *L*. *noguchii*, mustard: *L*. *santarosai*, pink: *L*. *weilii*, light blue: *L*. *borgpetersenii*. If no ST is assigned allelic profile is incomplete. Branches are coloured according to bootstrap support (500 bp) with increasing intensity. P = 2015, C = 2014, both UK; L = Lao PDR, 2014. (A) *glmU*, (B) *pntA*, (C) *sucA*, (D) *tpiA*, (E) *pfkB*, (F) *mreA*, (G) *caiB*
(PDF)
