# Supplementary material for: An Extended Multilocus Sequence Typing (MLST) Scheme for Rapid Direct Typing of Leptospira from Clinical Samples
Source: PLoS Negl Trop Dis. 2016 Sep 21;10(9):e0004996. doi: 10.1371/journal.pntd.0004996 (PMC5031427; doi:10.1371/journal.pntd.0004996)

S1 Fig. E-gel pictures of PCR products from first round and nested PCRs of selected *Leptospira* strains in different dilutions.

first round PCR

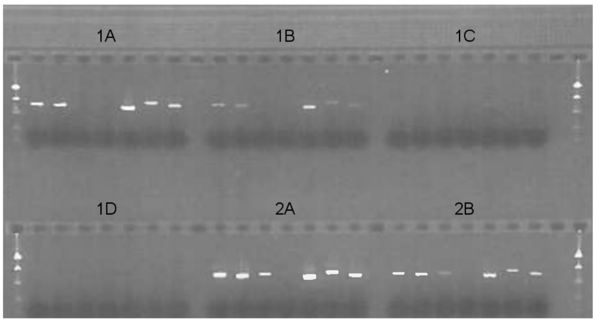

first round PCR

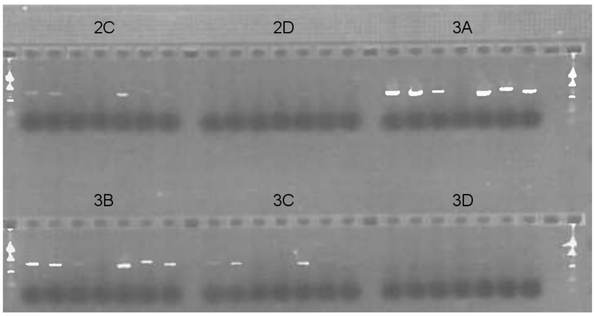

nested PCR

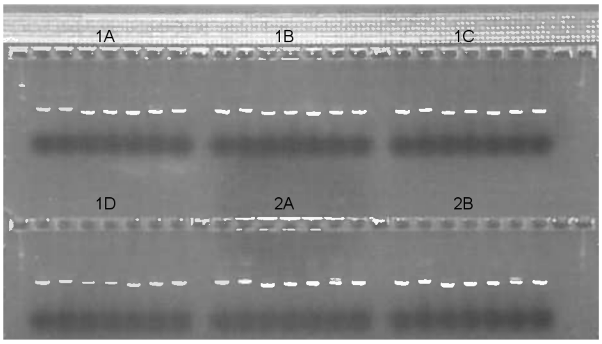

nested PCR

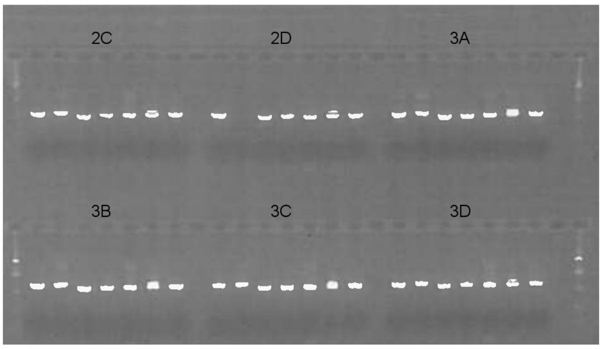

first roound PCR

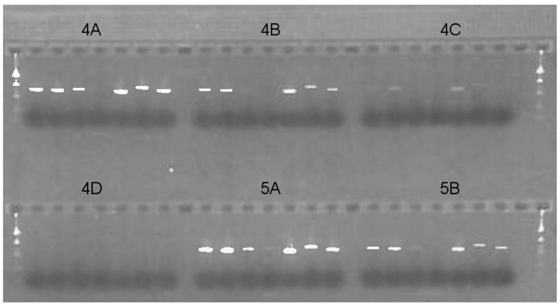

first round PCR

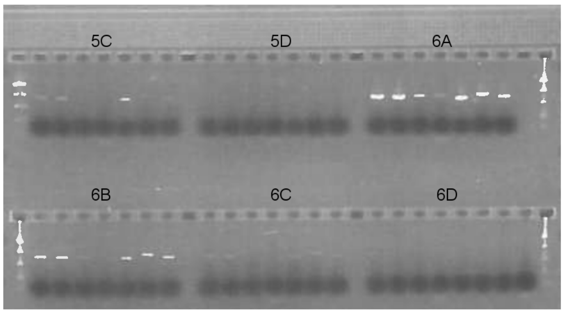

nested PCR

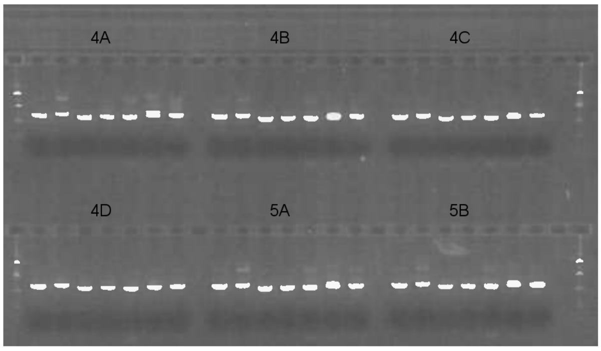

nested PCR

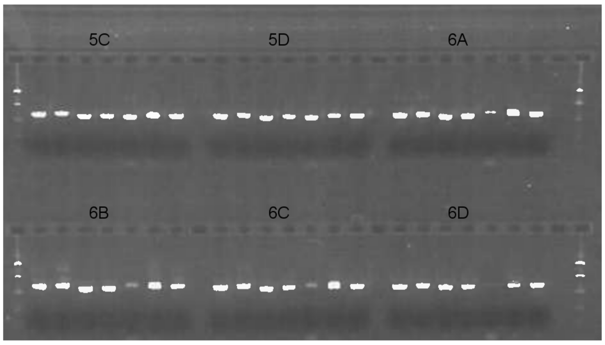

Supplement: S1 Fig — 1: Sari, 2: Wijnberg, 3: Hardjoprajitno, 4: Duyster, 5: Hond Utrecht IV, 6: Salinem. Dilutions (genomic copy numbers): A: 10E-2 (8000), B: 10E-3 (800), C: 10E-4 (80), D: 10E-5 (8). Dilutions are separated by one empty gel pocket. Each sample is applied in the following order: glmU-pntA-sucA-tpia-pfkB-mreA-caiB. DNA ladder size from top to bottom: 2000, 800, 400, 200,100 basepairs. (PDF) [file pntd.0004996.s004.pdf]
