# Supplementary material for: An Extended Multilocus Sequence Typing (MLST) Scheme for Rapid Direct Typing of Leptospira from Clinical Samples
Source: PLoS Negl Trop Dis. 2016 Sep 21;10(9):e0004996. doi: 10.1371/journal.pntd.0004996 (PMC5031427; doi:10.1371/journal.pntd.0004996)
Supplement: S2 Fig — Bar represents substitutions per site. Tips are labelled with sample_ST and coloured according to species: marine = L. interrogans, red: L. kirschneri, green: L. noguchii, mustard: L. santarosai, pink: L. weilii, light blue: L. borgpetersenii. If no ST is assigned allelic profile is incomplete. Branches are coloured according to bootstrap support (500 bp) with increasing intensity. P = 2015, C = 2014, both UK; L = Lao PDR, 2014. (A) glmU, (B) pntA, (C) sucA, (D) tpiA, (E) pfkB, (F) mreA, (G) caiB (PDF) [file pntd.0004996.s005.pdf]

S2 Fig. Phylogenetic trees including WHO recommended *Leptospira* serovar panel and clinical samples based on separate alleles.

A – *glmU* **B – *pntA*** **C – *sucA***

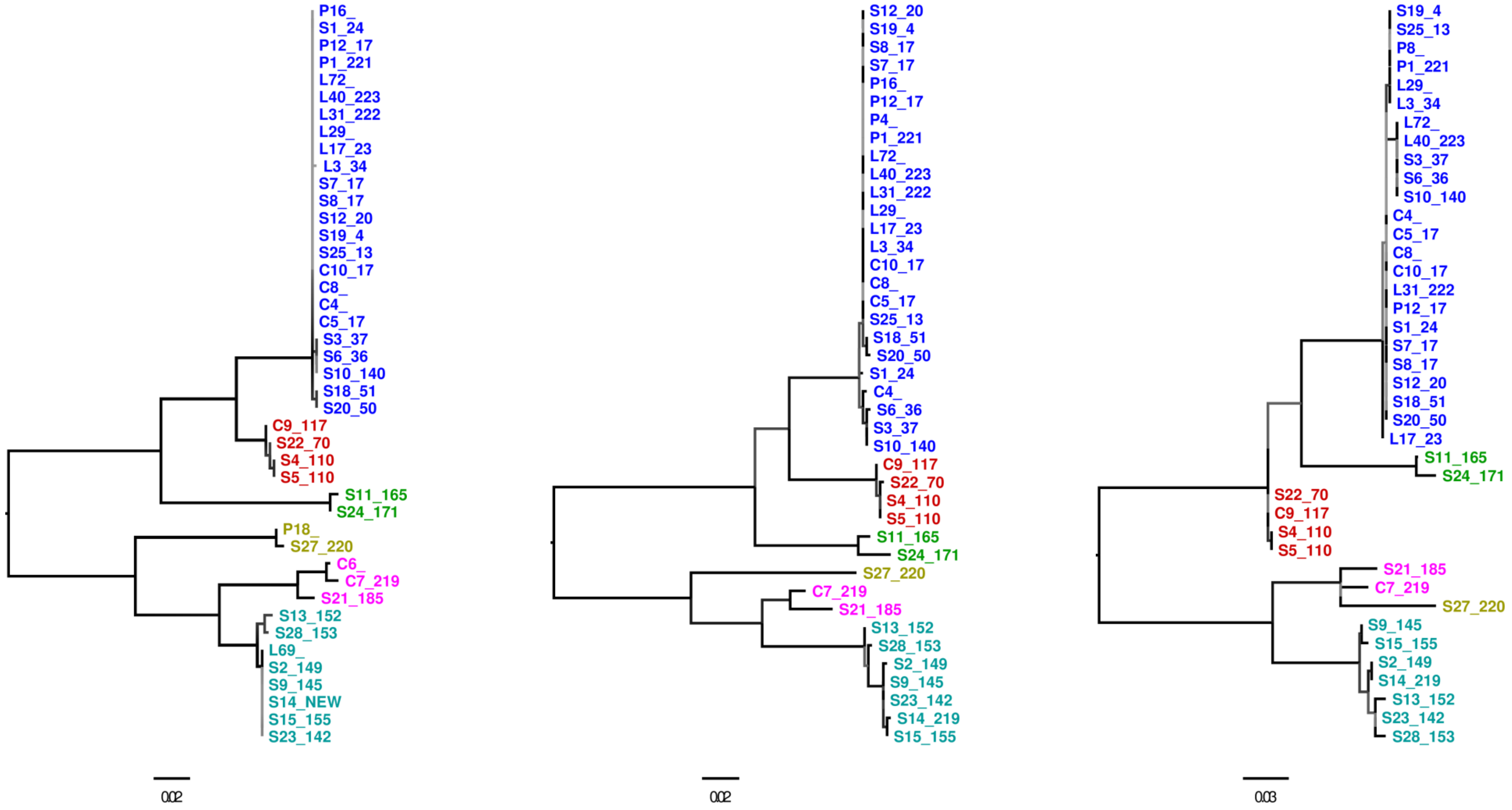

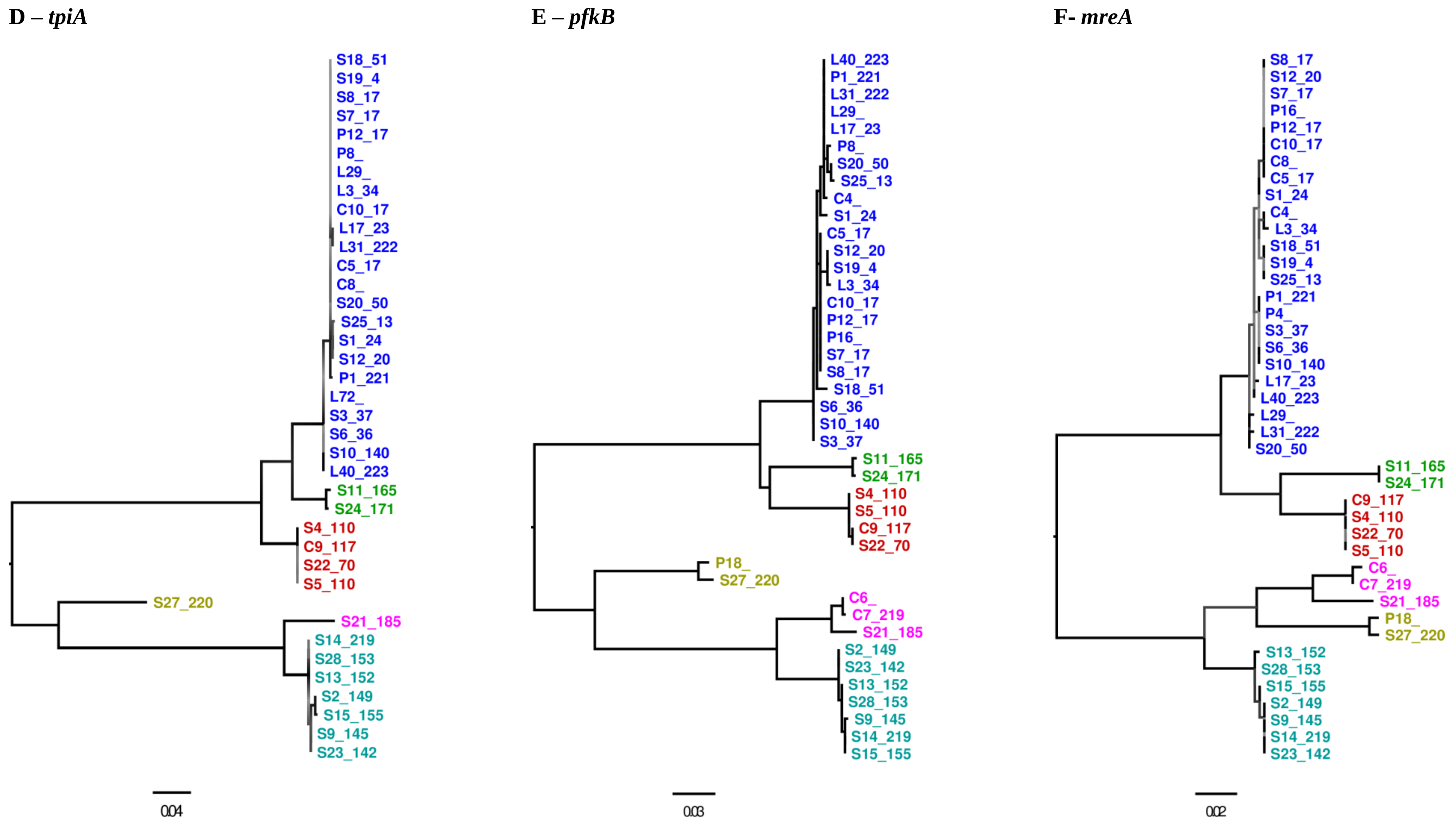

D – tpiA
S18_51
S19_4
S8_17
S7_17
P12_17
P8_
L29_
L3_34
C10_17
L17_23
L31_222
C5_17
C8_
S20_50
S25_13
S1_24
S12_20
P1_221
L72_
S3_37
S6_36
S10_140
L40_223
S11_165
S24_171
S4_110
C9_117
S22_70
S5_110
S27_220
S21_185
S14_219
S28_153
S13_152
S2_149
S15_155
S9_145
S23_142
0.04
E – pfkB
L40_223
P1_221
L31_222
L29_
L17_23
P8_
S20_50
S25_13
C4_
S1_24
C5_17
S12_20
S19_4
L3_34
C10_17
P12_17
P16_
S7_17
S8_17
S18_51
S6_36
S10_140
S3_37
S11_165
S24_171
S4_110
S5_110
C9_117
S22_70
P18_
S27_220
C6_
C7_219
S21_185
S2_149
S23_142
S13_152
S28_153
S9_145
S14_219
S15_155
0.03
F- mreA
S8_17
S12_20
S7_17
P16_
P12_17
C10_17
C8_
C5_17
S1_24
C4_
L3_34
S18_51
S19_4
S25_13
P1_221
P4_
S3_37
S6_36
S10_140
L17_23
L40_223
L29_
L31_222
S20_50
S11_165
S24_171
C9_117
S4_110
S22_70
S5_110
C6_
C7_219
S21_185
P18_
S27_220
S13_152
S28_153
S15_155
S2_149
S9_145
S14_219
S23_142
0.02

G - *caiB*

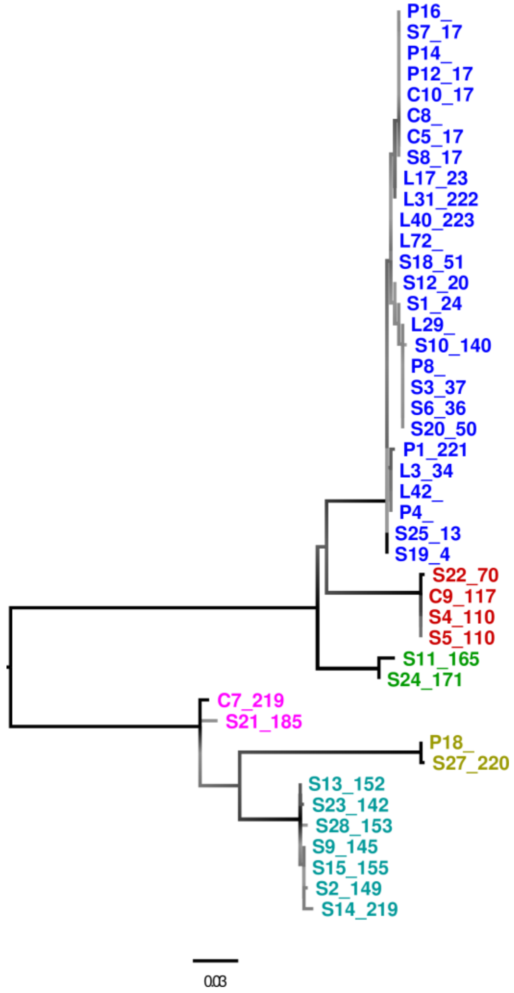
P16_
S7_17
P14_
P12_17
C10_17
C8_
C5_17
S8_17
L17_23
L31_222
L40_223
L72_
S18_51
S12_20
S1_24
L29_
S10_140
P8_
S3_37
S6_36
S20_50
P1_221
L3_34
L42_
P4_
S25_13
S19_4
S22_70
C9_117
S4_110
S5_110
S11_165
S24_171
C7_219
S21_185
P18_
S27_220
S13_152
S23_142
S28_153
S9_145
S15_155
S2_149
S14_219
0.03
